# Association between baseline clinical and imaging findings and the development of digital ulcers in patients with systemic sclerosis

**DOI:** 10.1186/s13075-019-1875-1

**Published:** 2019-04-15

**Authors:** S. Friedrich, S. Lüders, A. M. Glimm, S. G. Werner, G. Schmittat, G. R. Burmester, M. Backhaus, G. Riemekasten, S. Ohrndorf

**Affiliations:** 1 Department of Rheumatology and Clinical Immunology, Charité – Universitätsmedizin Berlin, Berlin, Germany; 2 Department of Radiology, Charité – Universitätsmedizin Berlin, Berlin, Germany; 3 Department of Gastroenterology and Rheumatology, Charité – Universitätsmedizin Berlin, Berlin, Germany; 4 Department of Rheumatology, Helios St. Johannes Klinikum Duisburg, Duisburg, Germany; 5 Department of Internal Medicine - Rheumatology and Clinical Immunology, Park-Klinik Weißensee, Berlin, Germany; 6 Department of Rheumatology and Clinical Immunology, University of Schleswig-Holstein, Lübeck, Germany

**Keywords:** Systemic sclerosis, Capillaroscopy, Color Doppler ultrasound, Fluorescence optical imaging, Raynaud’s phenomenon, Disturbed microcirculation, Digital ulcers

## Abstract

**Objective:**

Systemic sclerosis (SSc) can lead to ischemic complications such as digital ulcers (DUs). The aim of the study was to find predictors of DUs by clinical and new imaging methods.

**Patients and methods:**

All 79 SSc patients included in the study received a clinical, colour Doppler ultrasound (CDUS), fluorescence optical imaging (FOI) and capillaroscopy examination at baseline, and their capacity to predict new DU development was analysed in 76 patients at 12 months follow-up.

**Results:**

Twenty-two of 76 patients (28.9%) developed new ulcers during follow-up (diffuse SSc 48.1%; limited SSc 18.4%). Receiver operating characteristic (ROC) curve analysis revealed an area under the curve of 0.7576 for DU development, with a specificity of 87% and a sensitivity of 54.6% (*p* = 0.0003, OR = 8.1 [95%CI 2.5–25.6]) at a cut-off of ≥ 21 points (ACR/EULAR classification criteria for SSc). Capillaroscopy and CDUS had high sensitivity (100% and 95.5%) but low specificity (28.9% and 22.2%) for ulcer occurrence when used alone, but better specificity (46.3%) when combined (OR = 18.1 [95%CI 2.3–144.4]; *p* = 0.0004). Using FOI, fingers with pathologic staining had a higher risk for new ulcer development in the same finger (*p* = 0.0153). General future DU (i.e. DU also in other fingers) was associated with a missing FOI signal in the right digit III at baseline (*p* = 0.048).

**Conclusion:**

New imaging modalities can predict digital ulcer development in SSc patients with high sensitivity for capillaroscopy and CDUS and enhanced specificity when combined. A missing signal of FOI in the right digit III at baseline was associated with general future DU.

## Introduction

Systemic sclerosis (SSc) is an autoimmune connective tissue disease. The initial symptom in most patients is Raynaud’s phenomenon (RP), a condition characterized by the temporary reduction of blood flow to the fingers and toes (digits) due to a combination of reversible vascular spasms and irreversible alterations of the walls of blood vessels. Ultimately, systemic sclerosis leads to ischemic complications such as digital ulcers (DUs) and pitting scars (PS) in 50% of all SSc patients due to micro- and macroangiopathic changes in the vascular walls as well as progressive endothelial dysfunction [1–3]. These complications often result in severe pain, loss of function, inflammation and, occasionally, even amputation. Numerous investigators have attempted to improve the prediction of DU development, and certain risk factors, such as male gender, smoking, autoantibody profile, geographical factors, diffuse subtype, history of DU, early onset of Raynaud’s disease and early first non-RP, have been identified [4–13]. Other research focused on the role of microangiopathy (as presented in nailfold capillaroscopy) [14–20]. For example, in a multicentre study, it was found that the mean number of capillaries per millimetre in digit III of the dominant hand and clinical signs of severe digital ischemia at baseline have significant associations to new DUs during a 6-month follow-up [19].

Macrovascular changes are also present in SSc and linked to ischaemia: pathologic ultrasonography findings of the flow-mediated dilation of the brachial artery in patients with SSc were associated with the development of new DUs during 3 years of follow-up [20]. A higher risk of DU occurrence in a mean follow-up time of 53 months was demonstrated in patients with ulnar artery occlusion [21]. Colour Doppler ultrasonography (CDUS) represents a reliable tool to differentiate between primary and secondary RP when inspecting the proper palmar digital arteries (PPDA), the superficial arteries of the palms and the radial und ulnar arteries at the wrist level [3, 22]. Additionally, correlations of CDUS findings with microvascular damage have been reported [3].

In a previous study, our group confirmed a high prevalence of pathologically altered vessels in CDUS of the hands and fingers of patients with SSc. Moreover, a significant association between at least one pathologic PPDA and the presence of DU/PS in the same finger could be found (AUC 0.727). Furthermore, a shortened examination protocol (digits II to V of the right hand) was introduced to reduce the examination time [23, 24].

Fluorescence optical imaging (FOI) is a relatively new imaging technique, which has mainly been performed in the diagnostics of different arthritides. It depicts the distribution of the fluorophore indocyanine green (ICG) in the tissue of the hands and thereby visualizes the acral microcirculation. In patients with active joint inflammation, a strong signal enhancement of the fluorophore in the affected joints can be observed [25–27]. Recently, our group presented that low or even missing ICG staining was observed in the fingertips of SSc patients with no apparent RP as signs for a perturbed perfusion. These pathologic FOI findings showed associations to present DU and PS in the same finger [28].

This is the first study investigating associations between baseline clinical, capillaroscopy, CDUS and FOI findings and the development of digital ulcers in SSc patients over a 12-month follow-up period.

## Materials and methods

Ethical approval was granted by the local ethics committee of Charité – Universitätsmedizin Berlin (reference no. EA1/269/13). Signed informed consent was obtained from all patients.

Exclusion criteria were pregnancy, breast feeding, advanced renal disease (GFR < 30 ml/min), and hyperthyroidism.

LeRoy et al.’s definition of limited and diffuse cutaneous SSc was used to categorize SSc subtypes [29]. Patients with limited SSc sine scleroderma were included in the limited cutaneous SSc (lcSSc) subgroup.

At baseline, we conducted clinical, nailfold capillaroscopy, CDUS and FOI examinations in consecutively included in- and outpatients with SSc and collected data on their medical history and current symptoms. The American College of Rheumatology/European League Against Rheumatism (ACR/EUSTAR) classification guidelines were used to confirm the diagnosis and to assess the dissemination of SSc features in each patient [30, 31]. A 12-month follow-up questionnaire focussing on the development of new digital ulcers was planned.

All imaging examinations, which are described in detail below, were carried out at baseline and at an ambient temperature of about 21 °C to prevent thermal effects on perfusion.

**Nailfold capillaroscopy** was carried out using a USB device (Di-Li 970-O USB hand microscope Di-Li®-Lite). As specified by Cutolo et al. [32], each patient was categorized as having either a distinct SSc pattern (*early*, *active*, *late pattern*) or a non-SSc-specific pattern depending on their individual capillaroscopic features (capillary density, haemorrhages, megacapillaries, etc.).

**Colour Doppler ultrasonography (CDUS)** was conducted using the Mylab Twice device (Esaote, Genoa, Italy) and its linear array probe (9.1 MHz). Patients received a warm hand bath (ca. 38 °C) 5 min prior to the examination to facilitate vasodilation and prevent vasospasms induced by cold temperatures. Thirty-two vessels of the hands (20 proper palmar digital arteries [PPDA], 6 common palmar digital arteries, 2 superficial palmar arches, 2 ulnar arteries, and 2 radial arteries) were analysed and graded as either wide (*normal vessels*), narrowed or obliterated (*pathologically altered vessels*), as previously described [22–24]. A shortened examination protocol (the eight proper palmar digital arteries of the right hand’s digits II to V) showed similar associations with present digital ulcers at baseline [23, 24] and was assessed in regard to newly developed digital ulcers as well.

**Fluorescence optical imaging** was performed using the commercially available Xiralite X4 system (Xiralite GmbH, Berlin; Germany) in a standardized manner (using 0.1 mg/kg BW of the contrast agent indocyanine green [ICG], administered intravenously, image acquisition for 6 min and 360 images) [25, 26, 28, 33]. FOI analysis was conducted with XiraView® analytical software as stated previously, including a detailed description of the method. Inter- and intra-reader reliability was shown to be high for this method (*κ* = 0.834 and *κ* = 0.786 respectively) [28].

GraphPad Prism (Version 5.0 for Windows) commercial software was used for the statistical analyses. The univariate analysis Mann-Whitney *U* test was used for group comparisons, and Fisher’s exact and *χ*^2^ tests were used for comparing categorical variables as appropriate. Multiple linear regression (MLR) was conducted to confirm independent risk factors when appropriate. Odds ratios (OR) and their 95% confidence intervals (95%CI) are contributed below. A *p* value below 0.05 was considered significant. In the presented tables, a significance level < 0.05 is indicated by one asterisk (*), a level < 0.01 by two asterisks (**), and a level < 0.001 by three asterisks (***). Non-significant associations are marked “ns”.

## Results

After a mean of 11.7 (± 2.3) months, 76 subjects completed the envisioned questionnaire with items assessing the development of new digital ulcers (initial cohort *n* = 79, 3 drop-outs due to death or revoked cooperation).

### Subjects and their clinical characteristics

For baseline characteristics, see Table 1. During a mean follow-up period of 11.7 months (SD ± 2.3 months), 22 of 76 SSc patients (28.9%) reported the development of one or more digital ulcers. Patients with diffuse SSc developed DUs significantly more frequently than those with limited disease (48.1% versus 18.4%, respectively, OR = 4.1 [95%CI 1.5–11.7], *p* = 0.0087). They developed a mean of 2.5 (± 2.0) new DUs during this period.

**Table 1:**
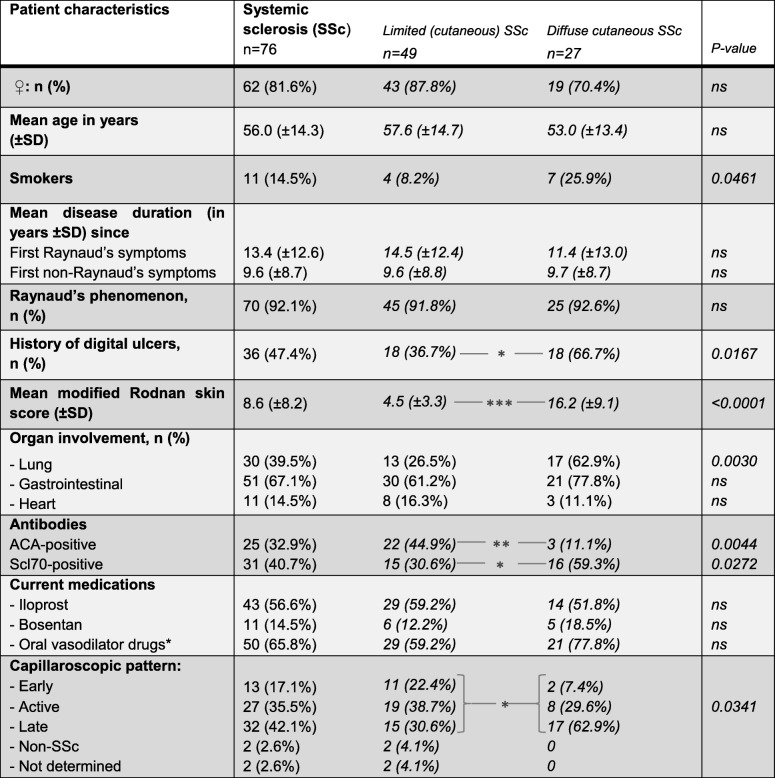
Baseline patient characteristics, including diagnosis, sex, age (± SD), Raynaud’s phenomenon, digital ulcers and nailfold capillaroscopy patterns as described by Cutolo et al. [32]

*ACE inhibitors, AT1 blockers, PDE5 inhibitors, calcium channel blockers, alpha1 antagonists

Italicized writing for subgroup analysis and corresponding p-values for improved comprehensibility of the data shown

### Clinical characteristics linked to new digital ulcers

**Fig. 1:**
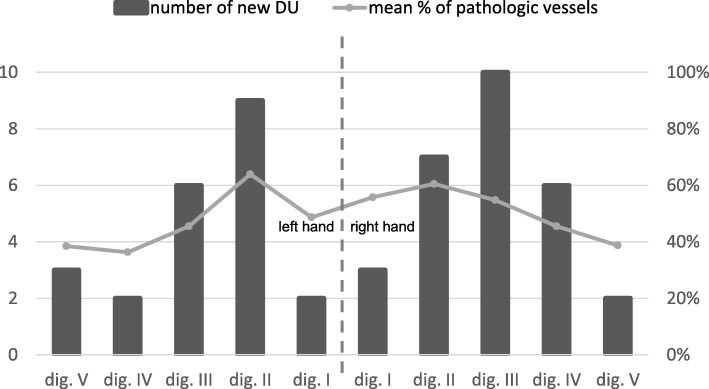
Number of newly developed digital ulcers per finger in patients with systemic sclerosis during follow-up (bars) compared to percentage of pathologically altered arteries per finger in all patients with systemic sclerosis (line)

Links between some known risk factors (e.g. known history of digital ulcers, diffuse subtype) and the development of digital ulcers were observed in the follow-up period. Table 2 shows the baseline characteristics of patients with or without new DU development during follow-up. Patients with diffuse (dcSSc) as well as limited cutaneous systemic sclerosis (lcSSc) developed new DUs during follow-up (mean 2.2 new DU per person [± 2.2] vs. mean 2.9 new DU [± 1.6]; ns). A summary of newly developed DUs per finger and hand is shown in Fig. 1 (bars). Patients treated with and without vasodilators developed similar numbers of new digital ulcers during follow-up.

**Table 2:** Characteristics of subjects with and without new digital ulcer (DU) development during follow-up

Patient characteristics at baseline *n* = 76^1^	Patients with new DUs (*n* = 22)	Patients without new DUs (*n* = 54)	*p* value	Odds ratio	95%CI; *p* value for cut-off
♀: *n* (%)	17 (77.3%)	45 (83.3%)	ns		
Mean age in years (± SD) at					
-Enrollment	56 (± 12.9)	56 (± 15.0)	ns		
-Raynaud’s onset	43 (± 14.4)	42 (± 16.4)	ns		
-Onset of first non-Raynaud’s symptom	45 (± 14.7)	46 (± 15.2)	ns		
Smoking					
-Current smokers, *n* (%)	5 (22.7%)	6 (11.1%)	ns		
-Mean pack years (± SD)	13.66 (± 16.38)	8.040 (± 13.4)	ns		
dcSSc, *n* (%)	13 (59.1%)	14 (25.9%)	0.0087	4.1	1.5–11.7; *p* = 0.0087
Mean modified Rodnan skin score (± SD)	13.4 (± 9.4)	6.7 (± 6.9)	0.0008	9.4^3^	3.0–29.2; *p* < 0.0001
ACR/EUSTAR score (± SD)	19.7 (± 4.0)	15.6 (± 4.1)	0.0004	8.1^4^	2.5–25.6; *p* = 0.0003
-Skin involvement					
-Thickening prox. of MCP, *n* (%)	12 (54.5%)	13 (24.1%)			
-Sclerodactyly, *n* (%)	9 (40.9%)	33 (9.1%)	0.0308	3.8^5^	1.3-10.8; *p* = 0.0154
-Puffy fingers, *n* (%)	1 (4.5%)	6 (61.1%)			
-Sine scleroderma	0	2 (3.7%)			
-Digital tip ulcers, *n* (%)	16 (72.7%)	20 (37.0%)	0.0058	4.5	1.5–13.5
-Pitting scars, *n* (%)	18 (81.8%)	22 (40.7%)	0.0020	6.5	1.9–22.0
-Teleangiectesia, *n* (%)	17 (77.3%)	43 (79.6%)	ns		
-Abnormal capillaroscopy, *n* (%)	22 (100%)	50 (96.2%)	ns		
-PAH, *n* (%)	6 (27.3%)	4 (7.8%)	0.0570	4.4	1.1–17.6
-Interstitial lung disease, *n* (%)	10 (45.5%)	16 (29.6%)	ns		
-Raynaud phenomenon, *n* (%)	22 (100%)	54 (100%)	ns		
-ACA pos., *n* (%)	8 (36.4%)	17 (31.5%)	ns		
-Scl70 pos., *n* (%)	9 (40.9%)	22 (40.7%)	ns		
-RNAP III pos., *n* (%)	2 (9.1%)	1 (1.9%)	ns		
Medications					
-Iloprost	14 (63.6%)	29 (53.7%)	ns		
-Bosentan	6 (27.3%)	5 (9.3%)	ns		
-Oral vasodilators^2^	18 (81.8%)	32 (59.3%)	ns		
Capillaroscopy pattern					
-Early, *n* (%)	0 (0%)	13 (24.1%)	0.0265	18.6^6^	1.1-326.4; *p* = 0.0035
-Active, *n* (%)	11 (50.0%)	16 (29.6%)			
-Late, *n* (%)	11 (50.0%)	21 (38.9%)			
-Non-SSc, *n* (%)	0 (0%)	2 (3.7%)			
-Not done, *n* (%)	0 (0%)	2 (3.7%)			

*CI* confidence interval, *dcSSc* diffuse limited cutaneous systemic sclerosis, *ns* not significant

^1^Baseline cohort: *n* = 79, 3 drop-outs

^2^ACE inhibitors, AT1 blockers, PDE5 inhibitors, calcium channel blockers, alpha1 antagonists

^3^Cut-off > 8 points

^4^Cut-off > 20 points

^5^Thickening proximal of MCP compared to other/no skin involvement

^6^Cut-off types, active and late

### ACR/EULAR classification criteria is a potential predictive tool for new digital ulcers

The 2013 ACR/EULAR classification criteria score for systemic sclerosis is based on a combination of typical SSc characteristics and diagnostic features. Although merely intended for classification purposes, the total score was not only significantly higher in patients who developed DU, but also showed good predictive value: Receiver operating characteristic (ROC) curve analysis showed an area under the curve (AUC) of 0.7576 for DU development, with a high specificity of 87.0% and a sensitivity of 54.6% (*p* = 0.0003; OR = 8.1 [95%CI 2.5–25.6]) at the cut-off value of ≥ 21 points. Of the single domains, a history of former skin lesions had the strongest association to future ulcers, followed by vasculopathy-related organ involvement (pulmonal arterial hypertension) and a more severe skin involvement (especially sclerodactyly and skin thickening proximal to the metacarpophalangeal joints compared to puffy fingers and no scleroderma) (see Table 2).

### Associations between capillaroscopy patterns and new digital ulcer development during follow-up

Only patients with an active or late pattern developed digital ulcers during follow-up (sensitivity 100%), but specificity was rather low (28.9%) since more than 70% of patients without new DUs presented an active or late pattern. The odds ratio (OR) for these patients to develop new digital ulcers was 18.6 [95%CI 1.1–326.4] (*p* = 0.0035). We found no significant relation between the late pattern alone and DU development.

**Fig. 2:**
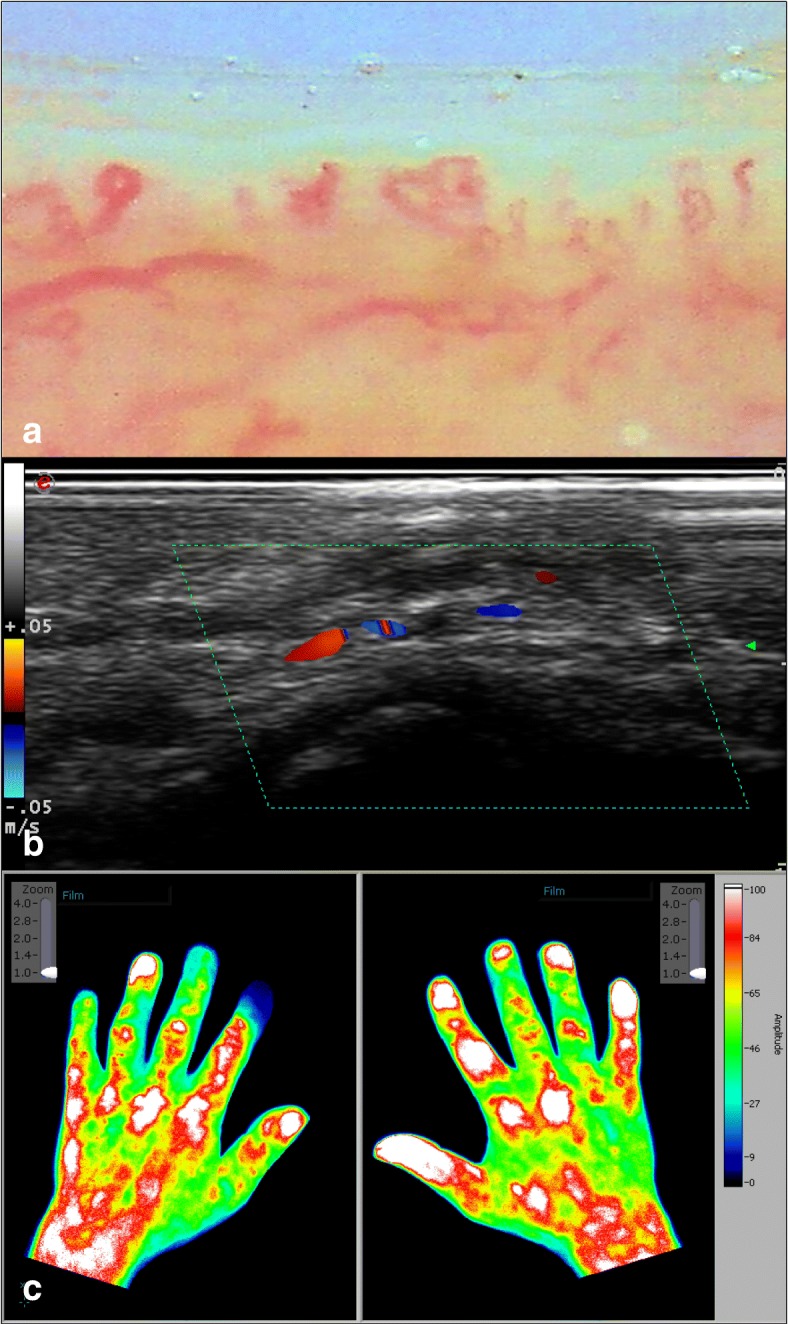
Exemplary depiction of the three imaging techniques: **a** Nailfold Capillaroscopy of a patient with diffuse SSc; the last capillary line is disorganized, the capillaries are rarefied (late pattern). **b** Colour Doppler ultrasonography shows a narrowed PPDA in a patient with diffuse SSc. **c** Fluorescence optical imaging in a patient with diffuse SSc; the index finger of the left hand shows a missing ICG enhancement as a sign of perturbed perfusion. SSc—systemic sclerosis; PPDA—proper palmar digital artery; ICG—indocyanine green

Of all fingers examined, reduced capillary density in the third digit of the right hand was most significantly associated with new DU development in general (*p* = 0.0266). Individual finger analysis showed that 11.2% of fingers with less than seven capillaries per millimetre developed digital ulcers compared to none of those with a normal capillary density at baseline (*p* < 0.0001; OR 30.6 [95%CI 1.8–500.6]). An example of rarefied capillary density in a patient with diffuse SSc is shown in Fig. 2a.

### Significant association between pathological CDUS findings at baseline and new DU development

The total proportion of blood vessel abnormalities (i.e. narrowing or occlusion) detected by CDUS at baseline was significantly higher in patients who developed DUs during follow-up (mean 46.6% [± 24.6%] pathologic vessels) than in those who did not (mean 32.9% [± 20.2%]; *p* = 0.0211). A correlation between the overall number of pathologic PPDAs per finger and new DUs during follow-up was also observed (Fig. 1). Fingers with at least one blood vessel abnormality (narrowing or occlusion) were more likely to develop digital ulcers during follow-up (*p* = 0.0149; OR = 2.6 [95%CI 1.2–5.9]). Fingers with exclusively occluded vessels were even more prone to develop DUs (*p* < 0.0001; OR = 9.2 [95%CI 4.5–19.0]). One example of pathologic PPDA in a patient with diffuse SSc is given by Fig. 2b.

### Capillaroscopy combined with colour Doppler ultrasonography has high sensitivity and moderate specificity

Colour Doppler ultrasonography alone showed relatively good sensitivity (all vessels 90.9% vs. right hand digits II–V 95.5%) and specificity (25.9% vs. 22.2%), which was superior to that of capillaroscopy. With CDUS and capillaroscopy combined, the specificity values improved to 44.2% while maintaining a high sensitivity of 90.9%: Twenty of 22 patients with digital ulcers exhibited an active or late pattern in capillaroscopy and more than 20% pathologic vessels in CDUS. Patients testing positive by both methods were eight times more likely to develop digital ulcers during follow-up (OR = 7.9 [95%CI 1.7–37.5]; *p* = 0.0032). We used a shortened CDUS protocol [23] that focuses on digits II to V of the right hand in combination with capillaroscopy patterns. This method only slightly improved sensitivity and specificity (95.5% and 46.3%, respectively), but achieved a substantial increase in the OR of new DU development during follow-up (OR = 18.1 [95%CI 2.3–144.4]; *p* = 0.0004).

### Fluorescence optical imaging-based individual finger analysis is more specific in terms of risk stratification

About a quarter of patients with both normal and abnormal initial enhancement (IE), characterized by missing signals in at least one fingertip in the beginning of the FOI examination, developed digital ulcers during follow-up (28.6% vs. 25.6% respectively, ns). Thirty percent of patients with abnormal maximal distal distribution (MDD) and 24.5% of those with normal MDD at baseline developed new digital ulcers later (*p* = 0.704). Abnormal MDD in fluorescence optical imaging showed rather high specificity (84.1%) and low sensitivity (20.0%) in detecting patients with new DU. The patients with abnormal MDD displayed only a tendency for more frequent new digital ulcer development (OR = 1.3 [95%CI 0.3–5.9]; *p* = 0.704). The predictive values increased slightly when FOI results were combined with capillaroscopy patterns or CDUS, but this remained non-significant (for both specificity 86.05% and sensitivity 25%; OR = 2.1 [95%CI 0.50–8.5]).

Both univariate analysis and multiple linear regression (MLR) indicated that a missing initial signal in digit III of the right hand was a risk factor (independent) for general (also in other fingers) DU development (*p* = 0.032 and *p* = 0.048, respectively) at the individual level.

Individual finger analysis showed significant correlations between new DU development during follow-up and pathologic finger staining during initial enhancement (IE) as well as maximal distal distribution (MDD) in the same finger at baseline. New DU development occurred in 10.7% of fingers lacking an initial signal in the fingertip at baseline compared to 3.5% in those with normal fluorophore distribution (*p* = 0.0008; OR = 3.3 [95%CI 1.6–3.6]). DUs developed during follow-up in 19.2% of fingers with an overall absence of signals in the fingertip (abnormal MDD) at baseline compared to 5.4% of those with normal staining (*p* = 0.0153; OR = 4.2 [95%CI 1.5–11.8]).

An example of pathologic FOI in a patient with diffuse SSc is shown in Fig. 2c.

## Discussion

Digital ulcers are a common complication of systemic sclerosis and have a major impact on the quality of life in SSc patients. Further research is needed to identify patients with an increased risk of DU development in order to adjust treatment accordingly and to ultimately reduce DU occurrence.

To our knowledge, this is the first study investigating the potential of CDUS and FOI to predict the development of new digital ulcers in SSc patients.

Capillaroscopy is not only a valuable diagnostic tool, but also a useful tool for predicting the development of digital ulcers in patients with systemic sclerosis. Several studies found that active and late capillaroscopic patterns have a high sensitivity for predicting new DU, but rather low specificity: independent of the number of patients included in previous studies (*n* = 77 to *n* = 423), the sensitivity of advanced capillaroscopic patterns was found to be around 95% [9, 19], but the specificity of those patterns for new DUs was only 12% in a 6-month period [19] and 33% in a 3-year period [9]. In the present study, active and late capillaroscopic patterns showed quite comparable sensitivity (100%) and specificity (29%) levels.

Quantitative scores designed to improve the performance of capillaroscopy in the prediction of digital ulcers have also been proposed. The best results in untreated patients were achieved with the capillaroscopic skin ulcer risk index (CSURI). With a sensitivity of 93% and a specificity of 85%, CSURI showed a high predictive value for new or non-healing digital ulcers, albeit over a short follow-up of 6 months [4, 34, 35]. However, the fact that this risk index can only be used in patients with megacapillaries is a notable disadvantage of this method. As 29% of patients in the present study did not have megacapillaries detectable by capillaroscopy, we were unable to utilize the CSURI.

Overall, in a 12-month period, 28.9% of patients in the present study, especially those with diffuse SSc, developed new digital ulcers during follow-up. Our data also suggested that a high modified Rodnan skin score (mRSS) and a history of DUs were linked to the development of new digital ulcers, as previously described [28].

Interestingly, the 2013 ACR/EULAR classification criteria score for systemic sclerosis showed a strong association with the development of digital ulcers. This score was developed and validated for classification purposes and is not intended to measure disease severity. Therefore, this finding needs to be replicated in an independent cohort.

Although CDUS alone showed good sensitivity and low specificity, the combination of CDUS with capillaroscopy increased specificity while maintaining high sensitivity, especially when focussing on digits II–V of the right hand. Thus, the proposed reduced CDUS protocol focussing on digits II–V of the right hand [23] appears to be a sufficient predictive risk stratification tool for new DU that shortens examination time.

FOI generally showed a low sensitivity and high specificity for predicting the development of new DUs. However, only pathologic FOI findings in the third digit of the right hand had a significant association with the general development of digital ulcers in multi-linear regression analyses. Nevertheless, individual finger analysis showed promising results in regard to determining which specific fingers were at risk of future DU development. Up until now, FOI has been used to detect inflamed joints due to increased microcirculation. Therefore, this novel imaging technique might profit from an additional software tool focusing on decreased microcirculation.

There have been studies trying to identify potential soluble biomarkers such as suPAR (plasma-soluble urokinase plasminogen activator receptor) and EGFL7 (epidermal growth factor-like domain 7) for SSc-related complications [36, 37]. Further studies should try to combine soluble and imaging biomarkers in order to provide a more complete picture of predictors for digital ulcers in SSc patients.

Limitations of the study included the self-assessment of DUs and local ischemia by patients in telephone interviews and standardization issues related to this method. However, the patients were instructed to look for specific changes, and most of them were familiar with these kinds of complications because they had a history of DU. Most of the included patients were treated regularly at our facility and were thus able to provide more detailed information if necessary. Due to the limited number of possible examiners, the participants were known to the investigators performing the CDUS, FOI and capillaroscopy examinations; thus, they were not blinded to each patient’s clinical history. Any future’s study design should try to remedy this fact.

Another limitation of this study is that the patient population consisted of many participants with advanced SSc, who developed new DU despite Bosentan and Iloprost treatment. We did not control for potential changes in microvascular morphology and blood flow related to current treatment [24]. The FOI method is an invasive diagnostically tool requiring an i.v. catheter similar to contrast-enhanced MRI. Most patients already presented with an i.v. catheter due to i.v. treatment. However, there are diagnostic tools (e.g. CDUS, capillaroscopy, laser-based methods), which are non-invasive.

## Conclusions

In conclusion, we identified several risk factors for digital ulcers in our study. In addition to well-known clinical features associated with the development of DUs (e.g. elevated mRSS value or history of digital ulcers), we found that the composite score of the ACR/EUSTAR classification criteria is strongly associated with new DU development and should be further investigated. Our results regarding the predictive value of capillaroscopy patterns were comparable with previous results and could be improved by combining capillaroscopy with colour Doppler ultrasonography of the finger arteries. Here, we evaluated a short examination protocol [23] and found that it is sufficient to assess vasculopathy. The detection of a high percentage of pathologic blood vessels by this method was associated with the development of digital ulcers in a mean follow-up time of 12 months, and the percentage of 20% narrowed or occluded vessels was proposed as the predictive risk cut-off for DUs. Fluorescence optical imaging may be an effective tool to identify malperfused areas [28], but only pathologic findings in the third finger of the right hand showed a significant association with general new DU occurrence in the present study. Interestingly, a reduced capillary density in the third digit of the right hand also proved to be an independent risk factor for future DUs in other fingers. This is similar to the results of the multicentre videocapillaroscopy (CAP) study in which a significant association between the third digit of the dominant hand and new DU development was observed [19]. This suggests that further research into these findings is needed on a larger scale.

In summary, this prospective study offers first insight into the potentials of two new imaging techniques, CDUS and FOI, as tools for the prediction of digital ulcers in SSc patients.

## Abbreviations

95%CI: 95% confidence interval
ACR/EUSTAR: The American College of Rheumatology/European League Against Rheumatism
AUC: Area under the curve
CDUS: Colour Doppler ultrasonography
dcSSc: Diffuse cutaneous SSc
DUs: Digital ulcers
FOI: Fluorescence optical imaging
ICG: Indocyanine green
IE: Initial enhancement
lcSSc: Limited cutaneous SSc
MDD: Maximal distal distribution
MLR: Multiple linear regression
mRSS: Modified Rodnan skin score
OR: Odds ratios
PPDAs: Proper palmar digital arteries
PS: Pitting scars
ROC: Receiver operating characteristic
RP: Raynaud’s phenomenon
SSc: Systemic sclerosis
